# Bibliography

**Published:** 1899-05

**Authors:** 


### Bibliography.

DEGENERACY: ITS CAUSES, SIGNS, AND RESULTS. By Eugene S. Talbot, M.D., D.D.S., Fellow of the Chicago Academy of Medicine, Member of the Chicago Academy of Sciences, etc. With One Hundred and Twenty Illustrations. London, Walter Scott, Ltd.; Charles Scribner's Sons, New York, 1898.

This work of Dr. Talbot is, in the estimation of the reviewer, by far the most satisfactory of any that have preceded it from his prolific pen. From the first page to the last it is of profound interest to the thinker who studies effect back to cause, and for the moralist it abounds in a multitude of facts that will aid him in arriving at just conclusions.

To many, who look askance whenever a work upon degeneracy is mentioned, this book may be regarded as simply one to be avoided as a dull accumulation of facts upon which crude conclusions have been based. No greater mistake could be made. The facts are given, it is true, but they are largely permitted to carry, by their own force, to legitimate conclusions. It would seem impossible to lay down this book without a positive conception that the human race is, without exception, born to degeneracy. That "the sins of the parents have been visited upon the children" through countless generations, until all, from the most cultivated to the most debased, are but modifications of inherited tendencies. It leads further to the conclusion that man, in the generic sense, is to a large degree irresponsible for his actions. This does not accord with the legal or religious idea, and in some sense is repugnant to all right-thinking minds, but there is this compensating thought, in this connection, that while humanity may degenerate through heredity, it can also advance by the same law, and this can best be accomplished by a thorough understanding of causes that have led to this degeneracy, and, thus building upon a gradual and ever-ascending scale, ultimately will obliterate the physical and moral ancestral defects. That this cannot be accomplished in one generation is fully demonstrated in this book, but that they can be removed is proven in the history of all civilizations. Were it otherwise, progress would be impossible. The deformed and the depraved, made so by defective knowledge or absolute ignorance, we may always have with us, but in a lesser degree as intelligence expands. It is in this direction that the work of the author should accomplish its best results.

It has been prepared with a view to interest those for whom it was especially intended, and is, therefore, divested of dry technicalities and replete with original views and impressive facts derived from various well-known authorities. These are of deepest interest, and frequently of a startling nature, as they demonstrate vividly the difficulties mankind labors under in the evolutionary process to a higher life.

The author says in his preface, " The work has been written with a special intention of reaching educators and parents. With this object, it has avoided laying stress on any one cause of degeneracy and ignoring factors which produce it and are aggravated by it. The *doctrinaire* reformer will here find no support for any limited theory. While it does not pretend in the slightest degree to give all the details of degeneracy, it attempts to lay down general principles for practical purposes in a way that permits their application to the solution of sociologic problems."

The heredity problem is summed up in a quotation the author gives from Luys, which is worth giving in full, as it practically covers the whole trend of thought upon this subject: "Heredity governs all the phenomena of degeneracy with the same results and the same energy as it controls moral and physical resemblances in the offspring. The individual who comes into the world is not an isolated being separated from his kindred. He is one link in a long chain which is unrolled by time, and of which the first links are lost in the past. He is bound to those who follow him, and to the atavic influences which he possesses; he serves for their temporary resting-place, and he transmits them to his descendants. If he comes from a race well endowed and well formed, he possesses the characters of organization which his ancestors have given him. He is ready for the combat of life, and to pursue his way by his own virtues and energies. But, inversely, if he spring from a stock which is already marked with an hereditary blemish, and in which the development of the nervous system is incomplete, he comes into existence with a badly balanced organization; and his natural defects, existing as germs, and in a measure latent, are ready to be developed when some accidental cause arises to start them into activity."

To show the scope in part of this admirable work, the contents include a very full "Introduction," "The Stigmata of Degeneracy," "Heredity and Atavism," "Consanguineous and Neurotic Intermarriages," "Intermixture of Races," "Toxic Agents," etc.

While the temptation comes to the reviewer to quote largely from the pages replete with facts, he is aware that this would give but an incomplete idea of the book as a whole. It must, therefore, be read in its entirety to compass the aims of the author, which seem to be to elucidate general principles and thus aid in advancing mankind to a higher standard physically and morally.

METHODS OF FILLING TEETH. An Exposition of Practical Methods which will enable the Student and Practitioner of Dentistry successfully to prepare and fill all Cavities in Human Teeth. By Rodrigues Ottolengui, M.D.S. Second edition. Two Hundred and Seventy-three Illustrations. The S. S. White Dental Manufacturing Company, Philadelphia; Claudius Ash & Sons, Limited, London, 1899.

This book has reached a second edition after the lapse of six years. This is not very creditable to the teaching force of the United States, for as a text-book for use in colleges it stands without a rival in its practical teaching of "methods of filling teeth." This slow absorption of an edition is, therefore, not due to a lack of positive merit, but is in all probability owing to a growing feeling on the part of students that they can get along without text-books during their student days in the practical branches, reserving the purchase of these to a future day that may never come.

This work of Dr. Ottolengui was fully reviewed in this journal upon its first appearance, and there is no reason now to extend or alter the opinion then held. While the reviewer does not regard with favor all the methods proposed, it remains true, in his opinion, that a careful following of all these, so clearly illustrated, will bring success even with a moderate degree of mechanical ability. While this latter talent is indispensable to great success in filling teeth, a very limited degree will suffice with careful and detailed teaching; while, upon the other hand, the mechanical genius may prove an entire failure, in his earlier years of practice, because of imperfect preliminary suggestions. This book, therefore, to both classes will be a valuable aid, and will prove a solution to them of many problems.

In reading this book, and considering the lapse of time intervening between the first and second edition, the thought naturally arises that this very fact demonstrates the marked advance in teaching. Forty years ago the ideas conveyed upon its pages would have been greedily absorbed by the then practitioners, and the book would have been regarded as a veritable treasure-house of practical ideas, to be procured at whatever cost to the individual. Now these same teachings are the daily experience of students, until they begin naturally to feel that all has been acquired that it is possible to learn in this branch of dentistry. This is to a large extent true, for it may be said that all the problems that once disturbed the fathers have been met and conquered.

Novelty in practice is not, therefore, to be found upon the pages of this book, but the manner of treatment of the various topics, coupled with a gracefulness of style, gives character to every description, claiming the attention of the reader.

While the book is complete in its practical details, it is measurably deficient whenever the author steps beyond the line to which he has limited his work. His etiology, limited as it is in the view of the writer of this, is singularly defective. It is not to be supposed that he is not familiar with the ideas extant, for he is probably one of the best informed upon the literature of the dental profession. This being so, why should he give this definition to abrasion: "*The cause is that the friction from the food causes a more rapid wearing away of dentine than of more resistant enamel.*" It is strange that any one should consider that food alone constitutes the principal factor in this destruction. Food acts but a minor part in the loss of tissue. The constant wear of attrition is a secondary factor, but the combination of attrition with acid conditions during sleep are unquestionably primal factors in destruction, the passage of food over occlusal surfaces occupying an inferior place in the deterioration of surface.

The etiology of erosion is not entered upon to any extent, and it would have been better had the author omitted all attempts in this direction, unless willing to extend his ideas to a more exhaustive treatment of the topic in hand.

In discussing materials for filling, the author can see but little good in tin or gold and tin combined. He seems absolutely ignorant of the value of the former, and gives but a paragraph to its use and nothing to its manipulation. This is certainly an error in a text-book. There is no place here for professional prejudices. The consensus of opinion of the best men in the dental profession, and of equal ability with the author, is that tin should never be permitted to drop out of use as a material for filling teeth. That it requires equal skill in manipulation with gold is admitted, but when thus placed in teeth it has a positive value, surpassing in many instances amalgam, and in teeth with hypersensitive dentine it is superior to both gold and amalgam. Tin and gold, as a material, is practically condemned as of no value. This is equally inconceivable in view of the experience, both at home and abroad, with this combination.

The main defect in the book seems to be that it is confined too much to the author's own ideas. These are, as has been stated, of positive value, but confining his method of treatment entirely to cohesive gold leads the untrained to the impression that there is no value in the use of non-cohesive gold, and the author intensifies this impression by stating, "In my own experience I have never discovered any special use for non-cohesive gold," and then gives a page of negative results. It is a waste of words to decry the value of this character of gold. To those who by long practice became familiar with its value the neglect of the author to explain the methods of use will seem worthy of decided censure and, in their estimation, would deservedly condemn the book. This would be an unfortunate conclusion. The true teacher must be many-sided and capable of broad explanations, but the author is right in confining his work mainly to those methods he had practically tested. While this is true, it places limitations upon the value of the book to the older practitioners and, possibly, may be, to that extent, an injury to the coming generation of dentists.

Criticism might be extended here and there throughout the volume, but this would only serve to call attention to slight defects in an otherwise carefully prepared work. It is thought, however, that the author would strengthen faith in the production with some by removing much of personal prejudice apparent in this volume from the third edition, when that should appear, a prejudice seemingly felt against all methods except those demonstrated to be of value in his own practice.

The book is presented with the care as to details so well known in all the production of the S. S. White Dental Manufacturing Company.

